# Food Insecurity and Anaemia Among a Tertiary Student Cohort: A One‐Centre Cross‐Sectional Study in Ghana

**DOI:** 10.1155/anem/1085315

**Published:** 2026-11-01

**Authors:** Patrick Adu, Ebenezer Tawiah, Israel Baffour Addo, Ernest Mensah, Samuel Tweneboah-Koduah, Redeemer Nana Fabea Addae, Prosper Junior Awittor, Irene Esi Amponsah

**Affiliations:** ^1^ Department of Medical Laboratory Sciences School of Allied Health Sciences University of Cape Coast Cape Coast Ghana ucc.edu.gh; ^2^ Anaemia and Haemoglobinopathy Research Group Medical Laboratory Diagnostics Graduate Programme Department of Medical Laboratory Science University of Cape Coast Cape Coast Ghana ucc.edu.gh

**Keywords:** anaemia, food accessibility, food insecurity, food insecurity questionnaire, Sub-Saharan Africa

## Abstract

**Background:**

Food insecurity (FI) is a significant global issue impacting well‐being and may affect academic performance among students. In Ghana, inflation‐induced high food prices and diminished purchasing power may exacerbate anaemia and FI among vulnerable populations.

**Aim:**

This study assessed the prevalence of FI and its relationship with anaemia among university students.

**Materials and Methods:**

This one‐centre cross‐sectional study used the validated food security questionnaires to assess FI among 385 full‐time undergraduate and postgraduate students. Additionally, haemoglobin level was estimated with the Hemocue Hb 201+ analyser to determine anaemia. Multivariate regression analysis was performed to evaluate relationships between FI and anaemia and other sociodemographic variables.

**Results:**

Respectively, 78.2% (301/385) and 67.5% (260/385) of the participants were FI on campus or at home. Relatedly, 63.7% (245/385) of the students were FI at both home and on campus; 14.5% were food secure at home but became FI on campus, whereas 3.9% were food secure on campus but became FI at home. Additionally, 27.8% (95% CI: 0.234–0.326) of participants were anaemic with significant disproportionate female representation (43.0% vs. 10.1% males, *p* < 0.001). Whereas male gender (78.1% vs. 58.5% females; *p* < 0.001), household income < 1000 GHS (*p* < 0.001) and basic parental education (*p* = 0.004) were significantly associated with FI at home, being a male (*p* = 0.003) or household income < 1000 GHS was significantly associated with FI on campus. Multivariate logistics analysis identified being a female as a significant risk factor of anaemia (aOR = 7.051, 95% CI: 3.818–13.023, *p* < 0.001); age groups, study type, regional differences, number of siblings, dependents, funding source, accommodation type and FI on campus did not show statistically significant associations.

**Conclusion:**

FI and anaemia are prevalent public health issues faced by the tertiary student cohort; intentional social intervention strategies are needed to address anaemia and improve food access.

## 1. Introduction

Food insecurity (FI) is the uncertain or limited availability of safe and nutritionally adequate foods to an individual or population [1]. It can vary in severity from mild food insufficiency to complete hunger and malnutrition. Despite notable advancements in global food production over the previous quarter‐century, FI remains a pressing global challenge. Approximately 795 million people worldwide still lack consistent access to sufficient, safe and nutritious food [2], equating to 8% to 20% of the global population [3]. Given the prevailing weak micro‐ and macroeconomics, wars and natural disasters, sub‐Saharan Africa (SSA) has been particularly affected by FI and accounts for nearly 60% of the global food‐insecure population [4].

In Ghana, FI is particularly severe in rural areas, which, generally, are agriculture‐based societies [5]. While most FI research has traditionally focused on households and rural communities, a growing body of literature highlights prevalence in higher education settings. A multi‐institutional study in the United States found between 14% and 59% of college students in various contexts experiencing FI during their academic careers [6]. Similarly, a study on FI among 15 to 24‐year‐olds in South Africa and Ghana revealed a high prevalence of FI, particularly severe FI [7]. However, food‐insecure students often remain unnoticed within the university systems. Many suffer in silence due to the stigma and shame associated with being classified as food insecure, resulting in a tendency to avoid seeking support from family or social welfare programmes. This concealment not only perpetuates the issue but also exacerbates its consequences. Empirical studies link FI among students to deteriorating mental and physical health, impaired academic performance, and reduced retention and graduation rates [2, 8]. A notable health‐related outcome frequently associated with chronic FI is anaemia (defined as reduced red blood cell counts and haemoglobin levels) [9]. Importantly, socioeconomic factors such as wealth status, residential setting and educational background are all significant determinants of FI and anaemia [10]. In developing countries like Ghana, unstable macro‐ and microeconomics and their attendant inflation lead to reduced purchasing power of the general populace and exacerbate FI [11–13]. Taken together, the issue of FI in developing economies disproportionately impacts vulnerable populations such as rural dwellers, women, children, adolescents and students [14, 15].

Despite increasing awareness, data on FI among students remain scarce in SSA. Previous studies in Ghana have demonstrated a high anaemia prevalence among university students [16], with a trend towards deterioration during the academic year [17]. However, the potential link between anaemia and FI among tertiary students remains under‐explored. This study sought to address this gap by evaluating the prevalence of FI and its association with anaemia among a cohort of students at a tertiary institution in Ghana. Taken together, by simultaneously exploring food access and factors associated with anaemia among a cohort of higher education students, our study conceptually demonstrates the intersectionality of SDGs 2 (no hunger), 3 (health and well‐being) and 4 (quality education).

## 2. Materials and Methods

### 2.1. Study Design and Setting

This observational cohort study assessed FI and anaemia among students at the University of Cape Coast (UCC), Ghana. Consenting participants were recruited at the beginning of the first semester (2022/2023 academic year) where haemoglobin levels were estimated. Subsequently, beginning from the middle of the semester, the Household Food Security Survey Module (HFSSM) was manually administered to participants to capture on‐campus food availability issues. During the inter‐semester break, the HFSSM was re‐administered through Google forms to capture food availability issues during the time participants were at home. Only individuals who consented for the initial haemoglobin measurement qualified to participate in the HFSSM questionnaire data collection. Moreover, the same cohort answered the HFSSM questionnaire both on campus and at home to enable us to capture the food security dynamics among the same cohort. The participant recruitment and data collection strategy is illustrated in Figure 1. The study was conducted at UCC, located in the Central Region of Ghana, a leading tertiary institution with a diverse student population enrolled in various undergraduate and postgraduate programmes. The recruited students provided data at two time points: Campus‐specific food access data were captured during the revision period just before the end‐of‐semester examination. Furthermore, the student cohort also completed a food security questionnaire during the first week of resuming a new semester to reflect food access at home. Blood sample for haemoglobin estimation was, however, undertaken once during the semester resumption period. As the university provides equal opportunities to students across all regions of Ghana, sampling the student population approximates FI and anaemia among young adults in Ghana. The study population comprised full‐time undergraduate and postgraduate students of UCC. Participation in the study was purely voluntary; no incentives were provided to participants, other than communication of the study outcome at the end of the research cycle. At the end of the research cycle, the research team met with the cohort and presented the results in an interactive presentation format where participants freely asked clarifying questions.

**FIGURE 1:**
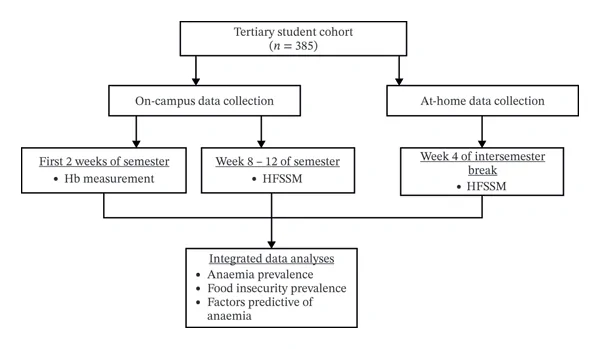
Schematic illustration of the data collection strategy for the food insecurity study among a cohort of University of Cape Coast students during the 2022/2023 academic year (Hb: haemoglobin; HFSSM: Household Food Security Survey Module).

### 2.2. Sample Size Calculation

At the time of the study, the student population of the University of Cape Coast was 78,485. The sample size was determined using Cochran’s formula: *n* = *Z* ^2^^∗^ *p*(1 − p)/*e* ^2^, where *n* = sample size, *Z* = Z‐value (1.96 for a 95% confidence level), *p* = estimated proportion of the population (assumed to be 50% or 0.5), *q* = 1 − *p* and *e* = margin of error (5% or 0.05). Using this formula, a sample size of 385 students was calculated, ensuring a 95% confidence level with a 5% margin of error.

### 2.3. Sampling Procedure

Our study used a nonprobability sampling procedure. Specifically, we used the consecutive convenience sampling technique to recruit our study cohort. The research team advertised the study to students at the halls of residence and lecture theatres for voluntary participation. Those who volunteered were directed to specific sampling sites where the study aims, duration and voluntary nature were explained to the prospective participants. Only those who gave written informed consent were enrolled in the study. Given the repeated sampling nature of the study, phone numbers of participants were collected as part of the initial data collection for the at‐home HFSSM questionnaire data collection. However, to maintain anonymity of the participants, the data were de‐identified after the exit session where the study outcomes were communicated to participants in an interactive discussion session.

### 2.4. Inclusion and Exclusion Criteria

All registered students of the university, both graduate and undergraduate students, were eligible for participation. However, non‐Ghanaian students were excluded, as the differences in currency exchange rates mean that they might have different economic strengths compared to students of Ghanaian origin. This study also excluded students with clinically diagnosed chronic health conditions (e.g., sickle cell disease, renal disease, or malaria within one month of the study period), as that could directly influence haemoglobin levels. Furthermore, pregnant students or those undergoing treatment for anaemia were also excluded in this study. Moreover, given the potential confounding influence of menstruation, during the participant recruitment entry stage, all females who self‐disclosed that they were menstruating or within 2 days post menses were also excluded.

## 3. Data Collection Procedures

The experimental protocols involved two main procedures: administering a structured questionnaire and measuring haemoglobin levels to assess anaemia.

### 3.1. Questionnaire

A structured questionnaire used for data collection was an adaptation of the validated six‐item U.S. HFSSM. The HFSSM [18] is a widely used tool that captures the degree of food access, and based on established thresholds, responses were scored and categorised into ‘food secure’ and ‘food insecure’ groups. From the scoring of the HFSSM [18], raw scores of 0–1, 2–4 and 5–6 are classified as high/marginal food security, low food security, and very low food security, respectively. In this study, the low food security (2–4 score) and very low food security (Score 5–6) were merged as food insecure, whereas Score 0–1 was classified as food secure. Each participant was required to complete it two separate times: when on campus and when at home during vacation, to capture food access on campus and at home, respectively. In addition to the food access, the questionnaire also gathered data on sociodemographic characteristics, dietary habits, socioeconomic status, academic level and living arrangements (e.g., on‐campus residence or off‐campus housing), and dietary preference (i.e., vegetarian, vegan, or omnivore). Socioeconomic status was evaluated by asking about sources of financial support, such as parental support, scholarships, or part‐time employment.

### 3.2. Haemoglobin Measurement

Haemoglobin levels were measured using the Hemocue Hb 201+ analyser (Hemocue, Sweden), a portable device that provides immediate and accurate readings of haemoglobin concentration. A finger‐prick blood sample was collected from each participant using sterilised lancets. The Hemocue analyser was calibrated before each session per the manufacturer’s instructions. According to the World Health Organisation (WHO), a sex‐specific haemoglobin threshold was used to classify anaemia: non‐anaemic: Hb ≥ 11.5 g/dL (females), Hb ≥ 12.5 g/dL (males). Participants were informed of their haemoglobin levels immediately after the test, and those with low haemoglobin levels were advised to seek medical consultation. Based on the WHO’s thresholds [9], the public health significance of anaemia at the population level was classified as normal (≤ 4.9%), mild (5.0%–19.9%), moderate (20.0%–39.9%), or severe (≥ 40.0%). Given that a previous study [17] within the same university demonstrated that the anaemia situation worsened during campus stay, we measured the haemoglobin levels of participants within the first two weeks to as much as possible to avoid the confounding influence of campus stay on the anaemia prevalence.

### 3.3. Data Analysis

Data were analysed using the Statistical Package for the Social Sciences (SPSS), Version 27.1 (IBM Corp., Armonk, NY, USA). The distribution of the data was explored using the Kolmogorov–Smirnov test. Descriptive statistics were used to summarise the sociodemographic characteristics of the participants, including age, gender, academic level and living conditions. The chi‐square test was employed to explore the relationships between FI and anaemia and other sociodemographic factors. Multivariate logistic regression was used to predict factors predictive of anaemia through adjusted odds ratio (aOR). In our logistic regression analysis, anaemia was the dependent variable, whereas participants’ demographics, funding source, and food security status (on campus and at home) were the independent variables. Moreover, the enter method was used in our regression analyses to ensure that all the predictors were in the analysis in a single step. All statistical testing was undertaken using the two‐tailed assumption, and a *p*‐value < 0.05 was considered statistically significant.

## 4. Results

The sociodemographic variables of the study participants are presented in Table 1. The majority were female (53.8%), aged 20–29 years (77.9%), and undergraduate students (95.3%). A significant proportion came from coastal regions (70.9%) and had parents with tertiary education (41.3%). Most participants had 4–6 siblings (56.1%). FI was widespread among study participants. On campus, 78.2% were food insecure compared to 67.5% experiencing FI at home, demonstrating a 10.7% increase in FI by virtue of being on campus. Moreover, 63.7% (245/385) of the students were food insecure at home and on campus.

**TABLE 1:** Demographic characteristics and food security status of a cohort of University of Cape Coast students during the 2022/2023 academic year.

Variable	Frequency (%)
Sex	
Female	207 (53.8)
Male	178 (46.2)
Age (years)	
17–19	72 (18.7)
20–29	300 (77.9)
30–39	13 (3.4)
Study type	
Undergraduate	367 (95.3)
Postgraduate	18 (4.7)
Region	
Coastal area	273 (70.9)
Forest	83 (21.6)
Savannah	18 (4.7)
College	
Education	25 (6.5)
CANS	62 (16.1)
CoHAS	81 (21.0)
HL	191 (49.6)
Missing#	26 (6.8)
Do you have any dependents?	
Yes	27 (7.0)
No	358 (93.0)
Parental education	
Basic	96 (24.9)
Secondary	120 (31.2)
Tertiary	159 (41.3)
Number of siblings	
1–3	123 (31.9)
4–6	216 (56.1)
> 6	46 (11.9)
Food security–campus	
Food secure	84 (21.8)
Low food secure	122 (31.7)
Very low food security	179 (46.5)
Food security–home	
Food secure	125 (32.5)
Low food secure	127 (33.0)
Very low food security	133 (34.5)
Household income (GHS)^∗^	
500–< 1000	165 (42.9)
1000–< 2000	93 (24.2)
> 2000	120 (31.2)
Source of funding	
Scholarship	10 (2.6)
Parent/guardian	348 (90.4)
Self‐funded	27 (7.0)

*Note:* #missing: participants did not state their respective college. HL: humanities and legal studies. The data are presented as frequencies *n* (%).

Abbreviations: CANS, College of Agriculture and Natural Sciences; CoHAS, College of Health and Allied Sciences.

^∗^Household income estimated in Ghana cedis: $1 is equivalent to GHS 10.42 at the time of the study.

Furthermore, the majority (56.1%) of the participants were from nonhealth‐related and natural sciences colleges.

Table 2 illustrates the prevalence of anaemia among the student participants. Out of 385 students, 27.8% were anaemic, with a significantly disproportionate female representation (43.0% females vs. 10.1% males; *p* < 0.001). Type of accommodation also showed a significant relationship (*p* = 0.032): Students living in university halls had higher anaemia prevalence (32.9%) than those in private (20.7%) or family housing (20.0%). Other variables such as age, income and region were not statistically significantly associated with anaemia, although trends revealed higher anaemia rates among students from coastal areas and lower‐income households.

**TABLE 2:** Anaemia status among a cohort of University of Cape Coast students stratified by sociodemographic parameters.

Variable	Total	Anaemia		*p*‐value
	*N* (%)	No 278 (72.2%)	Yes 107 (27.8%)	
Sex				< 0.001
Female	207 (53.8)	118 (57.0)	89 (43.0)	
Male	178 (46.2)	160 (89.9)	18 (10.1)	
Age (years)				0.233
17–19	72 (18.7)	47 (65.3)	25 (34.7)	
20–29	300 (77.9)	220 (73.3)	80 (26.7)	
30–39	13 (3.4)	11 (84.6)	2 (15.4)	
Study type				0.589
Undergraduate	367 (95.3)	264 (71.9)	103 (28.1)	
Postgraduate	18 (4.7)	14 (77.8)	4 (22.2)	
Region				0.200
Coastal belt	273 (70.9)	189 (69.2)	84 (30.8)	
Forest belt	83 (21.6)	64 (77.1)	19 (22.9)	
Savannah	18 (4.7)	15 (83.3)	3 (16.7)	
Household income^∗^				0.420
500–< 1000	165 (42.9)	121 (73.3)	44 (26.7)	
1000–< 2000	93 (24.2)	62 (66.7)	31 (33.3)	
> 2000	120 (31.2)	89 (74.2)	31 (25.8)	
Food security—campus				0.314
No	301 (78.2)	221 (73.4)	80 (26.6)	
Yes	84 (21.8)	57 (67.9)	27 (32.1)	
Accommodation type				**0.032**
University hall	228 (59.5)	153 (67.1)	75 (32.9)	
Private	140 (36.6)	111 (79.3)	29 (20.7)	
Family house	15 (1.3)	12 (80.0)	3 (20.0)	

*Note:* The data are presented as frequencies *n* (%); statistical significance at *p* < 0.05 is determined using the chi‐square test under two‐tailed assumptions; *p*‐values in bold indicate statistical significance at *p* = 0.05.

^∗^Household income is estimated in Ghana cedis: $1 is equivalent to GHS 10.42 at the time of the study.

Table 3 assesses the associations between demographic factors and FI at home and on campus. Female students were more food secure on campus (*p* = 0.003) and at home (*p* < 0.001). Students with household incomes greater than 2000 GHS had a strong inverse relationship with food security (*p* < 0.001 for both home and campus). Parental education was significantly associated with home‐based food security (*p* = 0.004), with students whose parents had only basic education being the most food insecure. Other factors like age, study type, region and number of siblings showed no statistically significant associations with FI, as well as coastal students and those with more (4–6) siblings. The majority of the participants (> 90.0%) did not have dependants, whereas only 2.6% were on scholarship. Further data disaggregation of the at‐home and on‐campus FI data (Supporting file S1) revealed that whereas 14.5% (56/385) of the students were food secure at home but became food insecure on campus (*p* < 0.001), 3.9% (15/385) were food insecure at home but became food secure on campus (*p* < 0.001).

**TABLE 3:** Demographic associations with food insecurity at home and on campus (among a cohort of University of Cape Coast students during the 2022/2023 academic year).

	Total	Food security at home		*p*‐value	Food security on campus		*p*‐value
		No 260 (67.5)	Yes 125 (32.5)		No 301 (78.2)	Yes 84 (21.8)	
Sex				**< 0.001**			**0.003**
Female	207 (53.8)	121 (58.5)	86 (41.5)		150 (72.5)	57 (27.5)	
Male	178 (46.2)	139 (78.1)	39 (21.9)		151 (84.8)	27 (15.2)	
Age (years)				0.486			0.719
17–19	72 (18.7)	45 (62.5)	27 (37.5)		56 (77.8)	16 (22.2)	
20–29	300 (77.9)	205 (68.3)	95 (31.7)		236 (78.7)	64 (21.3)	
30–39	13 (3.4)	10 (76.9)	3 (23.1)		9 (69.2)	4 (30.8)	
Study type				0.551			0.966
Undergraduate	367 (95.3)	249 (67.8)	118 (32.2)		287 (78.2)	80 (21.8)	
Postgraduate	18 (4.7)	11 (61.1)	7 (38.9)		14 (77.8)	4 (22.2)	
Region				0.063			0.321
Coastal belt	273 (70.9)	177 (64.8)	96 (35.2)		209 (76.6)	64 (23.4)	
Forest belt	83 (21.6)	60 (72.3)	23 (27.7)		70 (84.1)	13 (15.7)	
Savannah	18 (4.7)	16 (88.9)	2 (11.1)		14 (77.8)	4 (22.2)	
Household income^∗^				**< 0.001**			**< 0.001**
500–< 1000	165 (42.9)	129 (78.2)	36 (21.8)		138 (83.6)	27 (16.4)	
1000–< 2000	93 (24.2)	65 (69.9)	28 (30.1)		79 (84.9)	14 (15.1)	
> 2000	120 (31.2)	63 (52.5)	57 (47.5)		78 (65.0)	42 (35.0)	
Parental education				**0.004**			0.065
Basic	96 (24.9)	78 (81.3)	18 (18.8)		83 (86.5)	13 (13.5)	
Secondary	120 (31.2)	77 (64.2)	43 (35.8)		92 (76.7)	28 (23.3)	
Tertiary	159 (41.3)	99 (62.3)	60 (37.7)		118 (74.2)	41 (25.8)	
Number of siblings				0.927			0.465
1–3	123 (31.9)	97 (78.9)	26 (21.1)		78 (63.4)	45 (36.6)	
4–6	216 (56.1)	169 (78.2)	47 (21.8)		149 (69.0)	67 (31.0)	
> 6	46 (11.9)	35 (76.1)	11 (23.9)		33 (71.7)	13 (28.3)	
Do you have any dependents?				0.238			0.667
Yes	27 (7.0)	21 (77.8)	6 (22.2)		22 (81.5)	5 (18.5)	
No	358 (93.0)	239 (66.8)	119 (33.2)		279 (77.9)	79 (22.1)	
Source of funding				0.331			0.588
Scholarship	10 (2.6)	8 (80.0)	2 (20.0)		9 (90.0)	1 (10.0)	
Parent/guardian	348 (90.4)	231 (66.4)	117 (33.6)		270 (77.6)	78 (22.4)	
Self‐funded	27 (7.0)	21 (77.8)	6 (22.2)		22 (81.5)	5 (18.5)	

*Note:* The data are presented as frequencies *n* (%); statistical significance at *p* < 0.05 determined using the chi‐square test under two‐tailed assumptions; *p*‐values in bold indicate statistical significance at *p* = 0.05.

^∗^Household income estimated in Ghana cedis: $1 equivalent to GHS 10.42 at the time of the study.

Multivariate logistic regression analysis was used to explore possible risk factors of anaemia (Table 4). Female students showed increased adjusted odds of being anaemic compared to males (aOR = 7.05, *p* < 0.001). Other factors such as age group (aOR = 0.540, *p = 0.540*), undergraduate students (aOR = 1.678, *p* = *0.478*), residing location (aOR = 0.767, *p* = 0.767), family size (aOR = 1.469, *p* = 0.407), source of funding (aOR = 1.490, *p = 0.542*), accommodation (aOR = 1.581, *p* = 0.542), FI (aOR = 1.031, *p = 0.920*) and family dependents (aOR = 2.762, *p = 0.124*) were not statistically significant predictors of anaemia.

**TABLE 4:** Logistics regression analysis of risk factors for anaemia among a cohort of University of Cape Coast students during the 2022/2023 academic year).

Variables	B	Wald	*p*‐value	aOR	95% CI
Sex					
Female	1.953	38.931	**< 0.001**	7.051	3.818–13.023
Male	Reference				
Age (years)					
17–19	−0.617	0.424	0.515	0.540	0.084–3.452
20–29	−0.656	0.537	0.464	0.519	0.090–3.000
30–39	Reference				
Study type					
Undergraduate	0.518	0.503	0.478	1.678	0.401–7.023
Postgraduate	Reference				
Region					
Coastal belt	−0.265	0.138	0.710	0.767	0.189–3.106
Forest belt	−0.628	0.686	0.408	0.534	0.121–2.360
Savannah belt	Referent				
Number of children in the nuclear family					
1–3	0.229	0.214	0.643	1.257	0.477–3.317
4–6	0.385	0.687	0.407	1.469	0.591–3.651
> 6	Reference				
Do you have any dependents?					
Yes	1.016	2.362	0.124	2.762	0.756–10.092
No	Reference				
Source of funding					
Scholarship	0.341	0.113	0.736	1.407	0.193–10.259
Parent/guardian	0.399	0.312	0.577	1.490	0.367–6.046
Self‐financed	Reference				
Type of accommodation					
University hall	0.458	0.372	0.542	1.581	0.362–6.900
Private accommodation	−0.172	0.050	0.824	0.842	0.186–3.820
Family house	Reference				
Food insecurity on campus					
Yes	0.030	0.010	0.920	1.031	0.572–1.857
No	Reference				

*Note:* Statistical significance at *p* < 0.05 is determined using multivariate logistic regression analysis; B is the estimated coefficient/unstandardised regression weight of the regression model; Wald is the test statistic for the individual predictor variable derived from (B/SE)^2^. *p* values in bold indicate statistical significance at *p* = 0.05.

Abbreviations: aOR, adjusted odds ratio; CI, confidence interval.

## 5. Discussion

Anaemia is a significant multifactorial public health challenge in SSA. Unstable macro‐ and microeconomics in SSA, with its associated high inflation, hinder food accessibility, particularly among vulnerable groups like children, adolescents, reproductive‐aged women and students. Since nutritional deficits may cause anaemia, it is not far‐fetched that FI is an important factor to consider in the pathophysiology of anaemia. This observational study explored the association between FI and anaemia among a tertiary student cohort. Overall, there was high FI among the tertiary students, both at home (67.5%) and on campus (78.2%). Notably, more than six in 10 of the student cohort were stably food insecure irrespective of their at‐home or on‐campus environment. Moreover, whereas the overall anaemia prevalence (27.8%) was categorised as a moderate public health issue, data disaggregation showed that the female cohort demonstrated severe public health anaemia prevalence (43.0%). Taken together, anaemia and FI are each highly prevalent among the tertiary student cohort and require a concerted effort to improve nutrition among this vulnerable group.

The anaemia prevalence among the female cohort (43.0%) was indicative of a severe public health challenge that requires urgent attention and is in agreement with previous studies [17, 19]. This disproportionate female representation in anaemia prevalence among university students [16, 17] highlights the importance of data stratification and the need to explore sex‐specific interventional approaches in public health interventions. Anaemia may be caused by nutritional deficiency, inherited diseases, infection, malignancies or bleeding disorders; however, we speculate that chronic moderate to heavy menstrual blood loss may play a significant role in the high anaemia prevalence among the female participants [20]. We do not state this to rule out the possibility of compound causes to the anaemia reported herein, particularly in SSA settings, where multiple predisposition factors may coexist [21, 22]. However, since our study design did not consciously select for any of these conditions in male or female participants, and given the fact that we excluded actively menstruating females, we argue that chronic moderate to heavy menstrual blood loss, which is a uniquely evolutionary female experience, may be a major driver in the over‐representation of females in the anaemia data. It may be interesting for future studies to estimate actual menstrual blood losses and correlate these with blood count parameters to explore such a potential causal link.

With less than one‐third of the student cohort being food secure (whether at home or on campus), our data demonstrate that FI is a major concern among these students. Relatedly, FI demonstrated a 10.7% exacerbation (67.5% at home vs. 78.2% on campus) among the student cohort, further revealing a peculiar lack of food accessibility issues on campus. Our findings are not an oddity since previous studies have found similar high FI among university students in both high‐ and low‐income countries. For example, a previous study in a Ghanaian tertiary institution found 61.0% of participants [23] to be food insecure. In the United States, FI among university students has been estimated at 43.5% [24], 44.1% [25] and 48% [26], suggesting that food accessibility defies socioeconomic classifications of countries. In comparing FI prevalence data to the previous studies in the United States, we note that although the overall FI prevalence is comparable, FI severity differs considerably. Whereas low FI and very low food security were 31.7% and 46.5%, respectively, in this study, a similar study in the United States has recorded 17.1% and 30.9% for LFS and VLFS [26], demonstrating the need to not just consider the overall prevalence data. Interestingly, the female cohort in our present study was comparably more food secure, both at home (58.5% vs. 78.1% FI) and on campus (72.5% vs. 84.8% FI), compared to their male counterparts. This was despite the fact that the female cohort was disproportionately represented in anaemia prevalence data (43.0% vs. 10.1% males). Furthermore, our logistic regression analysis for factors predictive of anaemia also corroborated this finding since FI did not statistically significantly predict anaemia. However, female gender was associated with a sevenfold increased chance of anaemia (although they had a comparatively better food security metric). Taken together, although our study did not interrogate the nutritional value of the diets of these students, at face value, our findings point to a more complex relationship between apparent food accessibility, or the lack thereof, and anaemia. A previous study speculated that the high anaemia prevalence among tertiary students could be a function of FI [17]; a meta‐analysis by Moradi et al. also found a positive association between anaemia risk and FI [27]. However, based on our present data, we speculate that not only food accessibility, but also the quality and/or nutritional content of the diet, may be critical dimensions to consider in the nuanced anaemia—FI relationship. Relatedly, our regression analysis also showed that students in university halls of residence had an increased chance of anaemia. Although our present study did not collect food diversity scores to enable us to provide some contextual insights into this finding, future studies should explore this, given that students cook their own meals irrespective of whether in university‐provided halls or not. What is indisputably clear from our data is that students from lower‐income households or those whose parents had nontertiary education were significantly food insecure. Since educational attainment correlates positively with household earnings in Ghana [15, 28], our findings of high FI among the student cohort from homes within the lower economic class are not surprising and agree with previous research that identified gender, economic dependency and family structure as determinants of FI and nutritional health outcomes [29]. Prior research has highlighted the adverse impact of FI on student behaviour, well‐being and academic performance [6, 8]. Other researchers have also highlighted the adverse psychosocial impact of FI, particularly feelings of stigma and shame in college populations [1]. Future mixed‐methods studies which emphasise qualitative data through focus group discussion, ethnographic observations and participant interviews could help establish how much FI impacts academic performance and psychosocial dimensions of the academic journeys of these students. However, given the extent of FI revealed in this study and the previous University of Health and Allied Sciences’ study [23], we propose that Ghanaian tertiary institutions need to adopt a more proactive and systematic approach, like campus food pantries and meal vouchers and long‐term structural reforms, to address campus FI [8]. In any future pursuit of these interventions, we advocate that efforts must be made to maintain student dignity, protect privacy and reduce stigma to encourage uptake by these social interventions. However, given that the FI was recorded both at home and at school, efforts by national governments to improve the macro‐ and microeconomic environment to strengthen the economic muscles of families will provide long‐term solutions.

## 6. Limitations

This was a single‐centre study and may not necessarily be generalisable to other public or private tertiary universities in Ghana and across the SSA region since the socioeconomic characteristics of the student population may not be directly comparable. Furthermore, the observational nature of the study design limits causal inference, as we were unable to determine the directionality and/or temporality in the anaemia—food association. Moreover, given that the haemoglobin estimation was undertaken once, and at the beginning of the semester, we were unable to explore how the anaemia situation potentially changed during the semester. However, given that a previous study reported a worsening anaemia situation among students from the same university where the present study was undertaken [17], we speculate that dynamics in the anaemia situation are likely to be proportionate to the FI dynamics. Besides, given that we did not examine stool (for example, for occult blood loss and/or intestinal parasites) or urine for *Schistosoma haematobium* infection, we are unable to clarify the potential for these variables to have contributed to the anaemia prevalence data. Relatedly, the food security questionnaire is such that there was no direct measurement of parameters, as the data are self‐reported by the participants. Thus, we are unable to account for potential bias such as social desirability issues attendant to self‐reported data in questionnaire responses.

## 7. Conclusions

Although anaemia disproportionately affects females of our student cohort, females were comparatively more food secure (at home and on campus), suggesting a complex nonlinear relationship between mere food access and anaemia. Whereas public health measures implemented to address FI ought to target all students, given the high prevalence of FI, anaemia preventive measures should be more tailored to address the severe public health challenge related to females.

## Author Contributions

Conceptualisation: Patrick Adu; Methodology: Patrick Adu, Ebenezer Tawiah, Israel Baffour Addo, Ernest Mensah, Samuel Tweneboah‐Koduah, Redeemer Nana Fabea Addae, Prosper Junior Awittor, Irene Esi Amponsah; Software: Patrick Adu; data curation: Patrick Adu, Ebenezer Tawiah, Israel Baffour Addo, Ernest Mensah, Samuel Tweneboah‐Koduah, Redeemer Nana Fabea Addae, Prosper Junior Awittor, Irene Esi Amponsah; investigation: Patrick Adu, Ebenezer Tawiah, Israel Baffour Addo, Ernest Mensah, Samuel Tweneboah‐Koduah, Redeemer Nana Fabea Addae, Prosper Junior Awittor, Irene Esi Amponsah; validation: Patrick Adu, Ebenezer Tawiah, Israel Baffour Addo, Ernest Mensah, Samuel Tweneboah‐Koduah, Redeemer Nana Fabea Addae, Prosper Junior Awittor, Irene Esi Amponsah; formal analysis: Patrick Adu, Ebenezer Tawiah, Israel Baffour Addo, Ernest Mensah, Samuel Tweneboah‐Koduah, Redeemer Nana Fabea Addae, Prosper Junior Awittor, Irene Esi Amponsah: supervision: Patrick Adu; visualisation: Patrick Adu, Ebenezer Tawiah, Israel Baffour Addo, Ernest Mensah, Samuel Tweneboah‐Koduah, Redeemer Nana Fabea Addae, Prosper Junior Awittor, Irene Esi Amponsah; project administration; Patrick Adu; resources: Patrick Adu, Ebenezer Tawiah, Israel Baffour Addo, Ernest Mensah, Samuel Tweneboah‐Koduah, Redeemer Nana Fabea Addae, Prosper Junior Awittor, Irene Esi Amponsah; writing–original draft: Redeemer Nana Fabea Addae, Prosper Junior Awittor; writing–review and editing: Patrick Adu, Ebenezer Tawiah, Israel Baffour Addo, Ernest Mensah, Samuel Tweneboah‐Koduah, Redeemer Nana Fabea Addae, Prosper Junior Awittor, Irene Esi Amponsah.

## Funding

This study did not receive any public funding but was performed as part of the employment of the authors at the University of Cape Coast (UCC). The research was self‐financed by the co‐authors.

## Disclosure

The University of Cape Coast was not involved in the manuscript writing, editing, approval, or decision to publish this article. All authors have read and approved the final version of the manuscript. Patrick Adu had full access to all of the data in this study and takes complete responsibility for the integrity of the data and the accuracy of the data analysis.

## Ethics Statement

Ethical approval for the study was obtained from the University of Cape Coast Institutional Review Board (ethical clearance ID: UCCIRB/CHAS/2019/82), which reviewed the study’s objectives, methods and potential risks to participants. Ethical principles governing research with human subjects were strictly adhered to throughout the study. Participants were thoroughly informed about the study’s purpose and procedures. Their rights to voluntary participation, anonymity and the ability to withdraw from the study at any time were emphasised. Written informed consent was obtained from each participant before data collection. Confidentiality was maintained by limiting data access to the research team and ensuring that all identifiers were removed from the dataset.

## Conflicts of Interest

The authors declare no conflicts of interest.

## Supporting Information

Additional supporting information can be found online in the Supporting Information section.

## Supporting information


**Supporting Information** Food insecurity severity scores among a cohort of University of Cape Coast students were examined to situate the food insecurity dynamics at home and on campus (supporting file S1). Statistically significant differences characterised the food insecurity categorisation on campus and at home (*p* < 0.001). Although 67.5% of the students were food insecure at home, this increased to 78.2% when the students were on campus. Among the students who were food secure at home, 44.8% (56/125) became food insecure on campus. Relatedly, among the students who were food secure on campus, 17.9% (15/84) became food insecure at home. Moreover, when the food security severity scores were extrapolated, of the participants who were food secure at home, 28.0% and 16.8%, respectively, became low food security and very low food security on campus. Furthermore, of the participants who were food secure on campus, 15.5% and 2.4% became low food secure and very low food secure at home.

## Data Availability

The data that support the findings of this study are available from the corresponding author upon reasonable request.
