# Supplementary material for: Food Insecurity and Anaemia Among a Tertiary Student Cohort: A One‐Centre Cross‐Sectional Study in Ghana
Source: Anemia. 2026 Nov 1;2026:1085315. doi: 10.1155/anem/1085315 (PMC13725128; doi:10.1155/anem/1085315)
Supplement: Supplementary file 1 [file ANEM-2026-1085315-s001.docx]

**Supplementary file S1: Food insecurity severity scores among a cross-section of University of Cape Coast students.**

Food insecurity severity scores among a cohort of tertiary students were explored to evaluate severity at home and on campus. Statistically significant differences characterised the food insecurity categorization on campus and at home (p<0.001). Although 67.5% of the students were food insecure at home, this increased to 78.2% when the students were on campus. Among the students who were food secure at home, 44.8% (56/125) became food insecure on campus. Relatedly, among the students who were food secure on campus, 17.9% (15/84) became food insecure at home. Moreover, when the food security severity scores were extrapolated, of the participants who were food secure at home, 28.0% and 16.8% respectively became low food security and very low food security respectively on campus. Furthermore, of the participants who were food secure on campus, 15.5% and 2.4% became low food secure and very low food secure at home.

**Supplementary file S1: Disaggregation of the on-campus and at home food insecurity data**

|  |  | **Food security on Campus** | |  | p-value |
| --- | --- | --- | --- | --- | --- |
|  |  | **No [n = 301 (78.2)** | **Yes [n = 84 (21.8)** |  | **<0.001** |
| **Food security at Home** | **No [n = 260 (67.5)]** | 245 (81.4) | 15 (17.9) |  |  |
|  | **Yes [n = 125 (32.5)]** | 56 (18.6) | 69 (82.1) |  |  |
|  |  |  |  |  |  |
|  |  | **FS (n=84)** | **LFS (n=122)** | **VLFS (n=179)** | **<0.001** |
| **Food security at Home** | **FS (n=125)** | 69 (55.2)  (82.1)* | 35 (28.0)  (28.7)* | 21 (16.8)  (11.7)* |  |
|  | **LFS (127)** | 13 (10.2)  (15.5)* | 69 (54.3)  (56.6)* | 45 (35.4)  (25.1)* |  |
|  | **VLFS (n=133)** | 2 (1.5)  (2.4)* | 18 (13.5)  (14.8)* | 113 (85.0)  (63.1)* |  |

P values were calculated using chi-square after Bonferroni correction under a two-tail assumption; *indicates proportions calculated within campus score.
